# Effect of impurity resonant states on optical and thermoelectric properties on the surface of a topological insulator

**DOI:** 10.1038/s41598-017-04360-x

**Published:** 2017-06-21

**Authors:** Min Zhong, Shuai Li, Hou-Jian Duan, Liang-Bin Hu, Mou Yang, Rui-Qiang Wang

**Affiliations:** Guangdong Provincial Key Laboratory of Quantum Engineering and Quantum Materials, School of Physics and Telecommunication Engineering, South China Normal University, Guangzhou, 510006 China

## Abstract

We investigate the thermoelectric effect on a topological insulator surface with particular interest in impurity-induced resonant states. To clarify the role of the resonant states, we calculate the dc and ac conductivities and the thermoelectric coefficients along the longitudinal direction within the full Born approximation. It is found that at low temperatures, the impurity resonant state with strong energy de-pendence can lead to a zero-energy peak in the dc conductivity, whose height is sensitively dependent on the strength of scattering potential, and even can reverse the sign of the thermopower, implying the switching from n- to p-type carriers. Also, we exhibit the thermoelectric signatures for the filling process of a magnetic band gap by the resonant state. We further study the impurity effect on the dynamic optical conductivity, and find that the resonant state also generates an optical conductivity peak at the absorption edge for the interband transition. These results provide new perspectives for understanding the doping effect on topological insulator materials.

## Introduction

In the last decade, the discovery of topological insulators (TIs), such as Bi_2_Se_3_ and Bi_2_Te_3_, has triggered great interest in condensed matter physics due to their promising applications^1^. The topology property of the insulating bulk band in TIs protects metallic surface states, which are remarkably hallmarked by linear Dirac energy dispersion and spin-momentum locking nature^2–4^, immune from the backscattering off non-magnetic impurities. These surface states offer a unique platform to investigate the robustness of Dirac points against perturbations.

In order to manipulate the Dirac electronic properties, knowledge of the doping behaviors is of critical importance since the introduction of impurities, especially magnetic impurities, is a most natural way to reveal the topological properties of Dirac points^5^. Doping with nonmagnetic or magnetic impurities has been extensively applied^6–17^. They can lift the prohibited backscattering^6–10^ by opening an energy gap at the Dirac point or form resonant states^11–17^. Nevertheless, some works^14, 18–21^ demonstrated that the Dirac node remains immune from magnetic perturbations, challenging the gapped results^6–10^. To reconcile these conflicting claims, Black-Schaffer *et al*.^22^ theoretically suggested an interesting explanation that the resonance state induced by the non-magnetic potential can fill the energy gap generated by the magnetic potential. Subsequently, this interesting mechanism was verified experimentally^23^, where a dual nature of magnetic impurities was proposed. However, Sánchez-Barriga *et al*.^24^ addressed an opposite scenario about the resonance behaviors. They experimentally observed a large band gap (about 200 meV) in spite of doping with nonmagnetic impurity and attributed it to the strong resonant scattering processes. Thus, the behaviors of resonant state of impurities become ambiguous. Moreover, the formation of zero-energy resonance greatly destroys the pristine Dirac linear dispersion in TIs, which perhaps can act as an alternative approach to control the Dirac electron properties though lacking of backscattering, instead of opening a gap by magnetic doping. The appearance of the zero-energy resonance in recent experiments^13^ and numerical results^12^ challenges the existed theory^11^ and quantum impurity models^15, 17^ are put forward. One can notice that these impurity effects are usually discussed in the electronic band structure or energy spectrum of TIs. It is expected that the related transport properties, especially the thermoelectric properties which receives relatively less attention, would let one understand the impurity roles more deeply.

As we know, topological insulators are also good thermoelectric materials and so have attracted great interest^25–30^. The thermoelectric transports depend strongly on not only the thermal activation but also on the impurity scattering mechanism. By introducing disorders or holes in the Bi_2_Se_3_ material^31–33^, the thermoelectric figure of merit can be remarkably enhanced due to the contribution from the topologically protected conducting surfaces and the suppressed phonon thermal conductivity. Inversely, the thermoelectric transports can provide new insights into electronic transports and even extract the information not accessible for electrical conductance measurements. For instance, the thermoelectric power^26^ demonstrates the expected linear dependence in TIs. From the behavior of the transverse Peltier (or Nernst) conductivity at low temperatures, one can estimate the magnitude of the gap induced by time-reversal symmetry breaking and so be used to map the Berry phase structure^34^. Thermoelectric effects is also applied to detect the local spin-orbit interaction in graphene^35^. Employing the thermoelectric properties^36^, clear signatures of the topological surface states are extracted from the bulk states. In fact, the impurity-induced resonance leads to steep energy dependence of the electronic density of states, which would significantly affect the thermal properties. In graphene, the enhanced thermoelectric effect by resonant states was discussed^37^ and the information about impurity scattering can be extracted from the thermopower, either directly measured or extracted via Mott’s relation^38^.

In this paper, we focus on the influence of the impurity resonant state on electronic transport properties, where we calculate the dc and ac conductivities, the thermopower, and figure of merit. It is shown that although the scalar impurity cannot lead to the perfect backscattering, it can induce the resonant state and particle-hole asymmetry through skew scattering. The remarkable thermoelectric fingerprints manifesting impurity resonance are obtained. This paper is organized as follows. In Sec. II, a theoretical model is provided and the formula for thermoelectric coefficients are derived with impurity averaged Green’s function. We discuss the static dc transports in Sec. III and the dynamic ac transports in Sec. IV. A short summary is given in the last section.

## Model and Method

We consider a TI surface doped with impurities, which are described as point-like potentials randomly distributed at the position **r**_*n*_ and expressed in the framework of classic theory as ${V}_{im}({\bf{r}})={\sum }_{{{\bf{r}}}_{n}}(U{\sigma }_{0}-{\bf{M}}\cdot {\boldsymbol{\sigma }})\delta ({\bf{r}}-{{\bf{r}}}_{n})$. Here *U* is a scalar potential while **M **= *J*〈**S**〉 is a magnetic component associated with average impurity spin 〈**S**〉 and exchange coupling *J*, and σ〈*σ*_0_〉 is the vector of Pauli matrices (identity matrix). The Hamiltonian for the pristine surface state of **TI**s in the low-energy approximation is ${H}_{TI}={\sum }_{{\bf{k}}s}{c}_{{\bf{k}}s}^{\dagger }{H}_{TI}({\bf{k}}){c}_{{\bf{k}}s}$ with

1 $${H}_{TI}({\bf{k}})=\hslash {v}_{F}({k}_{y}{{\boldsymbol{\sigma }}}_{x}-{k}_{x}{{\boldsymbol{\sigma }}}_{y})-\mu ,$$

where ${c}_{{\bf{k}}s}^{\dagger }=({c}_{{\bf{k}}\uparrow }^{\dagger },{c}_{{\bf{k}}\downarrow }^{\dagger })$ is the creation operator of electrons with wave vector **k** = (*k*_*x*_,*k*_*y*_), and *v*_*F*_ is the Fermi velocity. Diagonalizing the Hamiltonian gives the energy dispersion ${\varepsilon }_{{\bf{k}}\gamma }=\gamma \hslash {v}_{F}|{\bf{k}}|-\mu$ with *γ* = ± referring to electron and hole bands, respectively.

The starting step for the calculation of transport properties is to obtain the impurity averaged Matsubara Green’s function G(**k**,*iω*_*n*_), which is given by the Dyson equation

2 $$G({\bf{k}},i{\omega }_{n})={G}_{0}({\bf{k}},i{\omega }_{n}){[1-{\rm{\Sigma }}(i{\omega }_{n}){G}_{0}({\bf{k}},i{\omega }_{n})]}^{-1}.$$

Here, the bare fermion Green’s function in 2 × 2 spinor space is ${G}_{0}({\bf{k}},i{\omega }_{n})=1/[i{\omega }_{n}-{H}_{TI}({\bf{k}})]$ and its matrix elements are defined as ${G}_{0}^{\sigma {\sigma }^{\text{'}}}({\bf{k}},i{\omega }_{n})=\langle {c}_{{\bf{k}}\sigma }|{c}_{{\bf{k}}{\sigma }^{\text{'}}}^{\dagger }\rangle$. The self energy ∑(*iω*_*n*_) contributed by impurity scattering is calculated by using of the *T*-matrix approach^15, 22, 39^, and in the Born approximation up to first order in impurity concentration *n*_*i*_ reads

3 $${\rm{\Sigma }}(i{\omega }_{n})={n}_{i}{[{\sigma }_{0}-{V}_{im}g(i{\omega }_{n})]}^{-1}{V}_{im},$$

which takes into account the multiple scattering of the electrons by a single impurity but neglects the effect of impurity correlations. In Equation (3), ${V}_{im}=U{\sigma }_{0}-{\bf{M}}\cdot {\boldsymbol{\sigma }}$ and

4 $$g(\omega )=\sum _{{\bf{k}}}{G}_{0}({\bf{k}},\omega )=\frac{1}{\mathrm{4(}\hslash {v}_{F}{)}^{2}}[\frac{\omega }{\pi }\,\mathrm{ln}(\frac{{\omega }^{2}}{{\Lambda }^{2}-{\omega }^{2}})-i|\omega |{\rm{\Theta }}({\rm{\Lambda }}-|\omega |]$$

with a cutoff energy Λ and a step function Θ(*x*). Proceeding the calculation for ∑(*iω*_*n*_), we can rewrite it in the Pauli matrix as

5 $${\rm{\Sigma }}(i{\omega }_{n})={{\rm{\Sigma }}}_{0}(i{\omega }_{n}){\sigma }_{0}+\sum _{j=x,y,z}{{\rm{\Sigma }}}_{j}(i{\omega }_{n}){\sigma }_{j},$$

where a scalar part ${{\rm{\Sigma }}}_{0}(i{\omega }_{n})=B[U-g(\omega )({U}^{2}-{M}^{2})]$ and magnetic parts ${{\rm{\Sigma }}}_{i}(i{\omega }_{n})=B{M}_{i},$ with $B={n}_{i}{\{{[1-g(\omega )U]}^{2}-g{(\omega )}^{2}{M}^{2}\}}^{-1}$ and *M* = |**M**| The self-energy is complex and energy-dependent, which will significantly modify the energy band structure or density of states as discussed below.

Substituting Eqs. (5) to (2), we rewrite the impurity averaged Green’s function as

6 $$G({\bf{k}},i{\omega }_{n})=\frac{1}{2}\sum _{\gamma =\pm }[1+{{\bf{n}}}_{{\bf{k}}\gamma }\cdot {\boldsymbol{\sigma }}]{G}_{\gamma }({\bf{k}},i{\omega }_{n}),$$

where we define a band-dependent Green’s function ${G}_{\gamma }({\bf{k}},i{\omega }_{n})={[i{\omega }_{n}+\mu -{{\rm{\Sigma }}}_{0}(i{\omega }_{n})-{\tilde{\varepsilon }}_{{\bf{k}}\gamma }]}^{-1}$ and an effective momentum unite vector ${{\bf{n}}}_{{\bf{k}}\gamma }=({\tilde{k}}_{y},{\tilde{k}}_{x},{{\rm{\Sigma }}}_{z}(i{\omega }_{n}))/{\tilde{\varepsilon }}_{{\bf{k}}\gamma }$ with ${\tilde{\varepsilon }}_{{\bf{k}}\gamma }=\gamma \sqrt{{{\rm{\Sigma }}}_{z}^{2}(\omega )+{(\hslash {v}_{F}\tilde{{\bf{k}}})}^{2}}$ and $\tilde{{\bf{k}}}=({k}_{x}+{{\rm{\Sigma }}}_{x}/\hslash {v}_{F},{k}_{y}+{{\rm{\Sigma }}}_{y}/\hslash {v}_{F})$. Note that ∑_z_(*iω*_*n*_) directly opens a band gap while ∑_0_(*iω*_*n*_) modifies the chemical potential *μ*.

With the impurity-averaged surface Green’s function derived above in hand, in this section we will calculate the thermoelectric coefficients. To proceed, we first define a current-current correlation function in the Matsubara representation ${{\rm{\Pi }}}_{ij}(i{{\rm{\Omega }}}_{n})=-{\int }_{0}^{\beta }d\tau {e}^{i{\Omega }_{n}\tau }\langle {T}_{\tau }{j}_{i}^{\dagger }(\tau ){j}_{j}\mathrm{(0)}\rangle$, where *τ* is the imaginary time and *T*_*τ*_ is the time ordering operator. In the one-loop approximation and neglecting vertex corrections^40^, one can write it as

7 $${{\rm{\Pi }}}_{ij}(i{{\rm{\Omega }}}_{n})=\frac{{e}^{2}}{\beta }\sum _{{\bf{k}},m}Tr[G({\bf{k}},i{\nu }_{m}){j}_{i}({\bf{k}})G({\bf{k}},i{{\rm{\Omega }}}_{n}+i{\nu }_{m}){j}_{j}({\bf{k}})].$$

where $\beta =1/{k}_{B}T$ with *T* temperature and Matsubara fermion frequency ${\nu }_{m}=(2m+1)\pi /\beta$. If one defines a spectral function *A*(**k**,*ω*) related to the Green’s function^41^ through $G({\bf{k}},i\omega )={\int }_{-\infty }^{\infty }d{\omega }_{1}\frac{A({\bf{k}},{\omega }_{1})}{i\omega -{\omega }_{1}}$, Equation (7) can be reexpressed as

8 $${{\rm{\Pi }}}_{ij}(i{{\rm{\Omega }}}_{n})=\frac{1}{{\rm{\Omega }}}\int \int d{\omega }_{1}d{\omega }_{2}[\frac{{n}_{F}({\omega }_{1})-{n}_{F}({\omega }_{2})}{{\omega }_{1}-{\omega }_{2}+i{\Omega }_{n}}]\,\sum _{{\bf{k}}}Tr[A({\bf{k}},{\omega }_{1}){j}_{i}({\bf{k}})A({\bf{k}},{\omega }_{2}){j}_{j}({\bf{k}})],$$

where we have used^40^ $\sum _{m}\frac{1}{(i{\nu }_{m}-{\omega }_{1})(i{\nu }_{m}+i{\omega }_{n}-{\omega }_{2})}=\frac{{n}_{F}({\omega }_{1})-{n}_{F}({\omega }_{2})}{{\omega }_{1}-{\omega }_{2}+i{\omega }_{n}}$ and the Fermi distribution function ${n}_{F}(\omega )={[exp([\omega -\mu ]/T)+1]}^{-1}$. For static transports Ω = 0, inserting in Equation (8) the electric current operator ${j}_{i}^{e}=-e\partial {H}_{TI}({\bf{k}})/\partial {k}_{i}$ or thermal current operator ${j}_{i}^{\kappa }=(\omega -\mu )\partial {H}_{TI}({\bf{k}})/\partial {k}_{i}$, one can find the linear response functions from the imaginary part of correlation function^40^ ${\mathrm{lim}}_{{\rm{\Omega }}- > 0}\frac{-1}{{\rm{\Omega }}}{\rm{Im}}\,[{{\rm{\Pi }}}_{ij}(i{{\rm{\Omega }}}_{n}\to {\rm{\Omega }}+i\delta )]$. As a consequence, when a temperature gradient is applied, all the thermoelectric coefficients can be computed with ${L}_{ij}^{n}$, given by

9 $${L}_{ij}^{n}=\frac{\pi }{2}{\int }_{-\infty }^{\infty }d\varepsilon (-\frac{\partial f(\omega )}{\partial \omega }){(\omega -\mu )}^{n}{K}_{ij}(\omega ),$$

with the response kernels ${K}_{ij}(\omega )=\sum _{{\bf{k}}}Tr[A({\bf{k}},\omega ){j}_{i}^{e}({\bf{k}})A({\bf{k}},\omega ){j}_{j}^{e}({\bf{k}})]$. Here we are interested in the longitudinal transports and the specific coefficients are, respectively, defined with the electric conductivity $\sigma ={e}^{2}{L}_{xx}^{0}$, the thermopower $S=-{L}_{xx}^{1}/eT{L}_{xx}^{0}$, and the figure of merit $ZT=\sigma {S}^{2}T/{\kappa }_{el}$ where ${\kappa }_{el}=[{L}_{xx}^{2}-{({L}_{xx}^{1})}^{2}/{L}_{xx}^{0}]/T$ is the electron heat conductivity and the phonon contribution is ignored for simplicity.

### Static DC transports

#### Impurity effect on transports for gapless topological surfaces

We begin our discussions on thermoelectric properties in the dc limit Ω = 0. Since the impurity scattering affects the electron transports by generating the extra self-energy, it is necessary to know the energy dependence of the self-energy. First, we discuss the case of doping only with finite scalar potential *U* but the magnetic potential *M* = 0.

**Figure 1:**
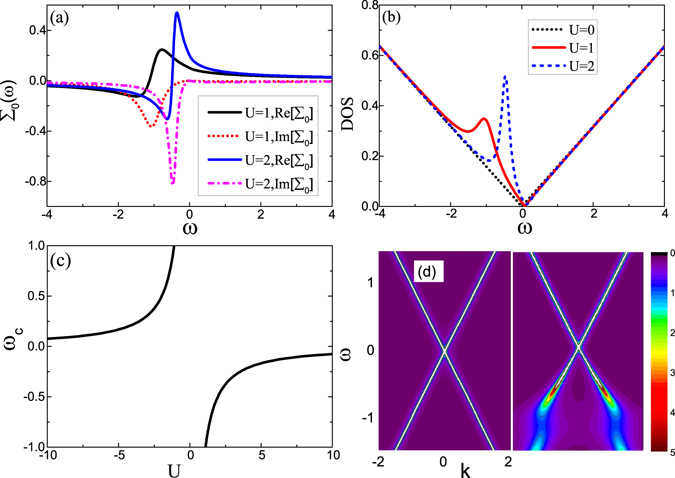
Evolution of (**a**) real and imaginary parties of impurity self-energies ∑_0_(*ω*) and of (**b**) DOS with impurity potential *U*, where a resonant state is developed remarkably. (**c**) The position of DOS resonant peak as a function of *U*. (**d**) The electron spectrum function *A*(**k**, *ω*) as functions of **k** and *ω* for *U* = 0 (left side) and *U* = 1 (right side). The other parameters are $\Lambda =300,T=0.01,M=0,\hslash {v}_{F}=1$, and *n*_*i*_ = 0.01.

In Fig. 1(a) we plot the real and imaginary parties of the impurity self-energy ∑_0_(*ω*) as a function of energy for different impurity potentials. Without *U* the self-energy ∑_0_(*ω*) vanishes. With finite *U* the most prominent feature for the self-energy is the energy dependence, especially in the vicinity of the Dirac point, where the imaginary part develops a dip which becomes remarkable with the increase of *U*. We know that the electron relaxation time is approximately proportional to the inverse of Im[∑_0_(*ω*)], so the enhanced dip with *U* implies the emergence of a resonance state. This point is verified in Fig. 1(b) where the density of state (DOS), defined as $\rho (\omega )=-\frac{1}{\pi }$ Im $Tr[{\sum }_{{\bf{k}}}G({\bf{k}},i{\omega }_{n}\to \omega +i{0}^{+})]$, is displayed as a function of energy. It is clearly visible that a pronounced resonance peak is caused by large *U* and its height is enhanced by *U*. The energy position *ω*_*c*_ of the resonant state is corresponding to the dip of Im[∑_0_(*ω*)], which is in turn determined by the real part of denominator of T matrix, i.e., Re[1 − *V*_*im*_g(*ω*_*c*_)] = 0. In Fig. 1(c), we illustrate the resonant position as a function of the impurity potential, from which one can find that with increase of *U*, *ω*_*c*_ quickly moves towards the Dirac point. For negative potential *U* < 0 the same resonance appears but at the side of positive energy. The result is in agreement well with ref. 11. It is interesting to compare the impurity-induced self-energy with phonon-induced one^42^ or spin inelastic-induced one^15, 43, 44^, where Im[∑_0_(*ω*)] is symmetry with respect to energy *ω* = 0 and Re[∑_0_(*ω*)] is asymmetry due to the electron-hole symmetry. But here, this symmetry is not abided by still, meaning that the impurity effect breaks the electron-hole symmetry. In Fig. 1(d), we plot the electron spectrum function $A({\bf{k}},\omega )=-\frac{1}{\pi }{\rm{Im}}Tr\,[G({\bf{k}},\omega +i{0}^{+})]$ as functions of **k** and ***ω***. For no disorder *U* = 0, the spectral function *A*(**k**,*ω*) is a simply Dirac delta function at *ω* = *ε*_**k***γ*_ and the energy dispersion shows the behavior of linear Dirac cone. The introduction of impurities shifts the energy band away from the Fermi level, leading to a large electron-hole asymmetry accompanied by the broaden spectrum. These unique features from the impurity effect will impact remarkably on the thermoelectric response as discussed below.

**Figure 2:**
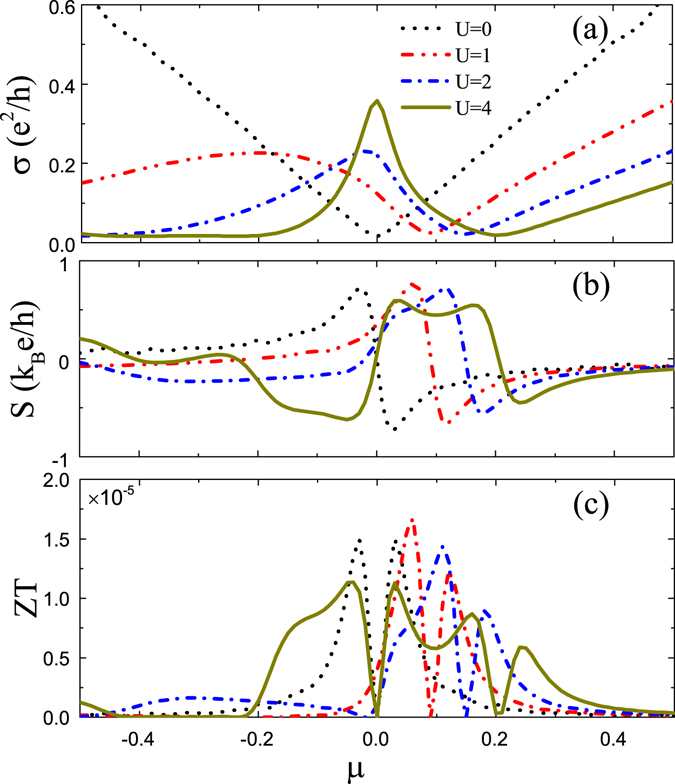
(**a**–**c**) Dependence on chemical potential *μ* of the electric conductivity σ, the thermopower *S*, and the figure of merit *ZT* for different scalar potential U = 0, 1, 2, 4. The other parameters are the same as in Fig. 1.

In Fig. 2(a–c) we plot the electric conductivity *σ*, the thermopower *S*, and the figure of merit Z*T* as a function of chemical potential *μ*, respectively. Figure 2(a) shows that the electric conductivity *σ* strongly depends on the impurity potential *U*. In the absence of impurities, *σ* exhibits a V-shaped structure, directly reflecting the Dirac linear dispersion, and the electron scattering happens only exactly at *ω* = *ε*_**k***γ*_. When impurity potential *U* is introduced, *σ* presents a non-monotonous dependence on *μ* owing to the formation of resonant state. As a consequence, there develops a resonance peak, which becomes narrow and high with the increase of *U*, accompanied by the minimum point of conductivity shifted to the high energy region. This scenario is greatly different from that in graphene^38, 45^ where the conductivity reaches a constant universal value 4e^2^/*πh* at low temperatures. In this situation, the position of resonance state is not only determined by the real part of 1 − *V*_*im*_g(*ω*) but also by its imaginary part. This makes the resonance position *ω*_*c*_ moves towards *μ* = 0 much faster. At the same time, the broaden bandwidth makes the electron scattering possible in an energy range of $\omega \in [{\varepsilon }_{{\bf{k}}\gamma }-Im{{\rm{\Sigma }}}_{0}(\omega )],[{\varepsilon }_{{\bf{k}}\gamma }+Im{{\rm{\Sigma }}}_{0}(\omega )]$. Even for electrons at *μ* = 0, there is large probability to scatter with the nearby electrons, producing the zero-point resonant peak of conductivity.

The Seebeck coefficient (or thermopower) *S* as a function of *μ* is displayed in Fig. 2(b), where a typical structure is presented for U = 0, namely, the thermopower is positive below the fermi level due to electrons dominating the transports while it is negative above the fermi level due to dominant hole traveling. At *μ* = 0, the thermopower vanishes, corresponding to the zero slope of the minimum point of conductivity. However, the existence of impurities changes this scenario by shifting the position of *S* = 0 to the high energy and even reversing the sign of *S* for large *U* (e.g., *U* = 4) in the low energy regime, exhibiting a broad hole-type region in pristine electron-type region. The former is attributed to the breaking of electron-hole symmetry while the latter is due to the contribution from the resonant peak, whose negative and positive slopes at either side corresponds to the sign of *S*. With the change of *S* sign, besides the zero point of *S* at the Dirac point, a new zero point of *S* appears, whose position is corresponding to the conductivity peak. At this point the conductivity contributed by electrons is just compensated by the conductivity due to holes.

In Fig. 2(c), we plot change of the figure of merit ZT with the impurity potential *U*. As *U* is increased, the lineshape of ZT varies remarkably because the Wiedeman-Franz law, which is an universal feature of conventional metals, is violated by the resonance state, i.e., $\frac{{\kappa }_{el}}{\sigma T}\ne \frac{{\pi }^{2}{k}_{B}^{2}}{3{e}^{2}}$. When strong impurity potential is introduced (e.g., *U* = 4), a most remarkable change is that there develops a double-dip structure, distinguished from the single dip for impurity-free case. The positions of two dips correspond to the position of *S* = 0.

**Figure 3:**
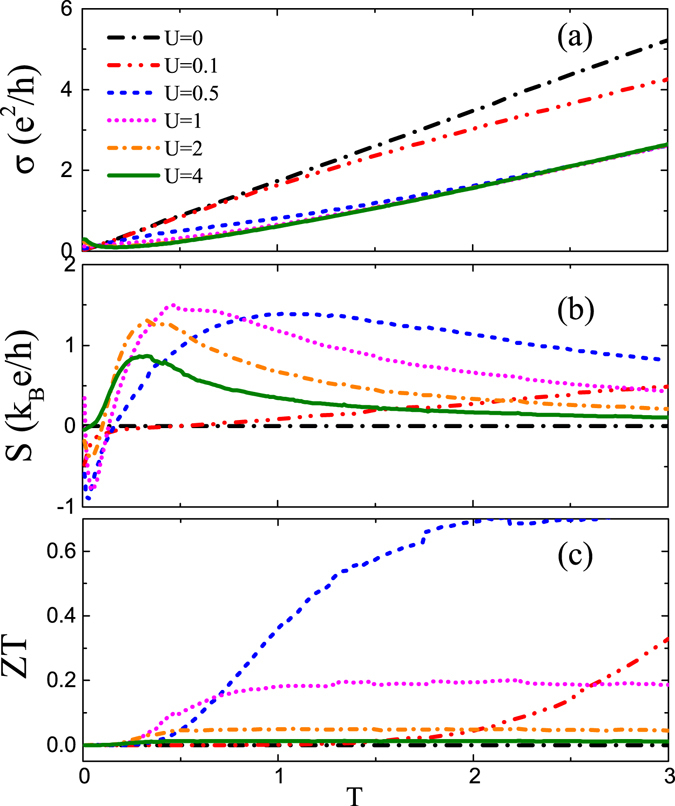
(**a**–**c**) The electric conductivity *σ*, the thermopower *S*, and the figure of merit *ZT*, respectively, at the Fermi level *μ* = 0 as a function of temperature *T* for various impurity potential *U* as indicated. The other parameters are the same as in Fig. 1.

For finite temperature, we in Fig. 3(a–c) plot the numerical results, respectively, for *σ*, *S*, and *ZT* at the Fermi surface *μ* = 0 as a function of temperature *T*. The electric conductivity σ is linear for undoped case *U* = 0, but they quickly deviates from this linear behavior for finite *U* with decreasing slop. By contrast, the thermopower *S* and the figure of merit *ZT* exhibit the non-monotonous dependence on the impurity potential *U* sensitively. For *U* = 0, the electron-hole symmetry leads to the vanishing *S* and *ZT* at the Fermi surface *μ* = 0. With the enhancement of *US* increases fast first at low temperatures and then decreases to a saturated value at high temperatures. Especially at *T*≈0, *S* appears a negative dip, which first becomes deeper and deeper and then is lifted to zero. This behavior is a consequence of the joint of the resonant state and the breaking of particle-hole symmetry. Figure 1(a) shows that with the increase of *U*, the conductivity resonance develops fist below the Fermi level and then moves to *μ* = 0 for large potential. From the Mott relation $S\propto -d\sigma (\mu ,T)/d\mu$, we can understand this behavior easily. The nonlinear dependence on *U* for *S* and *ZT* is significantly distinguished from the case of no resonance^26^, in which the impurity self-energy is treated simply as an energy-independent constant.

#### Impurity effect on transports for gapped topological surfaces

**Figure 4:**
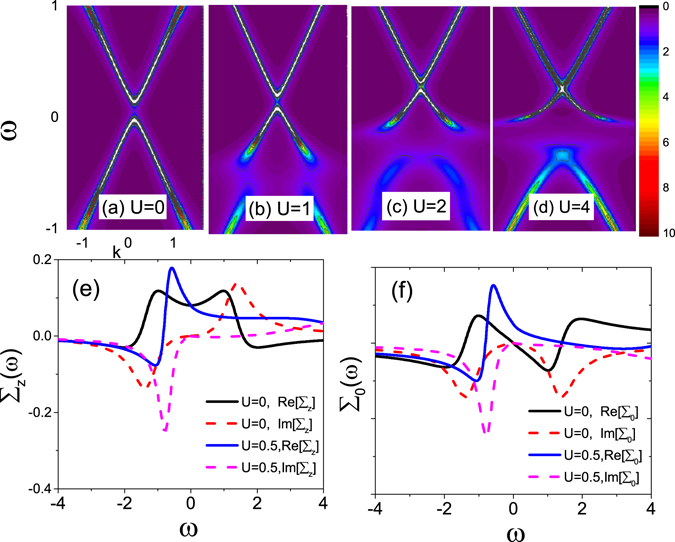
(**a**–**d**) Evolution of energy dispersion with impurity potential *U* = 0, 1, 2, 4 for gapped surface states. Dependence of (e) ∑_z_(*ω*) and (f) ∑_0_(*ω*) on *U*. The magnetic potential is chosen as *M* = 0.8 and the other parameters are the same as in Fig. 1.

Above discussions are for gapless topological surfaces. In this section, we extend them to the gapped band structure. If a surface state of TIs is doped with magnetic potential *M*, the electron dispersion of the surface state can open an energy gap at the Dirac point. In Fig. 4(a–d) we display the evolution of the band gap in spectral function *A*(**k**,*ω*) for increasing scalar potential *U* with fixed magnetic potential **M**. Without *U*, a distinct band gap appears, centered around the Dirac point, due to the massive energy band ${\tilde{\varepsilon }}_{{\bf{k}}\gamma }=\gamma \sqrt{{{\rm{\Sigma }}}_{z}^{2}(\omega )+{(\hslash {v}_{F}\tilde{{\bf{k}}})}^{2}}$. As *U* is introduced gradually, accompanied with the Dirac point of the band structure moving overall upwards, the band gap becomes narrower and narrower and finally disappears completely, seeing Fig. 4(d). The presence of an impurity resonance is characterized by the flattening and broaden part below the Dirac point, whose spectral edge is enough to fill the band gap completely for large *U*. The filling threshold is determined by the relative size between scalar potential and magnetic potential. For a typical TI Bi_2_Se_3_, the bulk energy gap is *Dc* = 0.3 eV. With this, *U* = 4 meV is needed in Fig. 4(d) to fill the band with gap of *M* = 0.8 meV, in the range of experiment parameters^23^. Notice that *U*-induced filling effect for gap is not through modifying the energy gap Δ directly but through the energy dependence of Re [∑_0_(*ω*)]. To clarify this point, we plot the dynamic self-energy ∑_z_(*ω*) and ∑_0_(*ω*) as a function of energy in Fig. 4(e–f), respectively. At the Dirac point ω = 0, the gap ${\rm{\Delta }}=|{\tilde{\varepsilon }}_{{\bf{k}}+}-{\tilde{\varepsilon }}_{{\bf{k}}-}|=2{\rm{R}}e{{\rm{\Sigma }}}_{z}\mathrm{(0)}=2{M}_{z}{n}_{i}$ is independent of *U*, so the bandgap is not modified by *U* through ∑_z_(*ω*), as shown in Fig. 4(e). As aforementioned, the real part of ∑_0_(*ω*) is to modify the chemical potential. It is noted that Re[∑_0_(*ω*)] in Fig. 4(f) always keeps asymmetric and Im [∑_z_(*ω*)] is symmetric until the scalar potential *U* is added, which transfers the weight from positive to negative energy to formate the resonance. As a consequence, Re [∑_0_(*ω*)] pushes the valence band edge moving toward high energy faster than the conducting band edge, thus effectively crowding out the band gap and even completely filling it. Similar mechanism is addressed for the gap filling by the spin inelastic scattering^44^.

**Figure 5:**
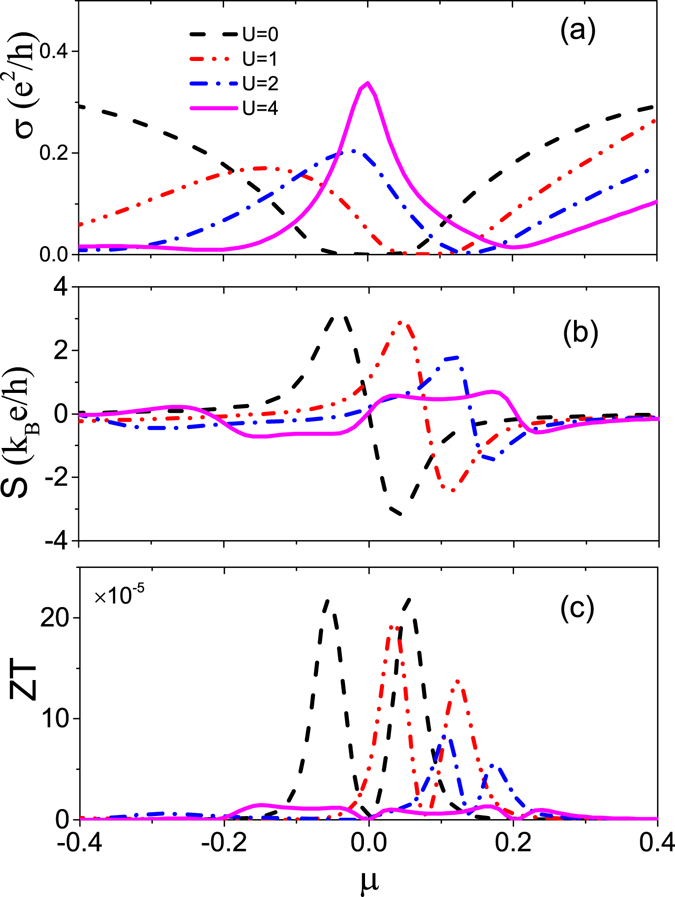
(**a**–**c**) The electric conductivity σ, the thermopower *S*, and the figure of merit *ZT* as a function of *μ* for *U* = 0, 1, 2, 4. We chose *M* = 0.8 and the other parameters are the same as in Fig. 1.

The magnetic impurity induced band gap is also visible in the conductivity σ, as shown in Fig. 5(a), where a zone of zero conductivity appears around the Dirac point (see black dashed line). Interestingly, as *U* is introduced, the resulting resonant structure at the low energy regime narrows the range of zero-conductivity zone, and for large magnitude (e.g., *U* = 4), the gap is completely filled and finite conductivity exists. In Fig. 5(b), we display the variation of thermopower *S* for different *U* values. Compared with gapless case in Fig. 3(b), the gaped conductivity can significantly enhance the magnitude of *S* though the pattern structure remains unchanged. Nevertheless, when the extra nonmagnetic potential *U* is added, the filling of the band gap causes the remarkable reduction in *S* size, and for large *U*, e.g., *U* = 4, *S* restores the gapless situation. In Fig. 5(c) we present the development of the figure of merit *ZT* with gap filling. Similarly, the fast reduction of ZT by the increase of *U* characterizes the gapped-to-gapless transition induced by the impurity resonant state. For large *U*, *ZT* also restores the double-dip gapless case. Therefore, the thermoelectric coefficients are sensitive to the gap filling or the competition between scalar and magnetic potentials.

If one rises the temperature *T*, the conductivity gap is easily smeared out by the joint contribution of the resonant state and thermal excitations. The dependence of the thermoelectric coefficients on temperature resembles to the gapless case as in Fig. 3.

### Dynamic AC transports

In this context, we consider the optical conductivity *σ*(Ω) in finite frequency, which can be probed by reflectivity experiments on the sub-THz to mid-IR frequency range. σ(Ω) gives the absorption spectrum for light with frequency Ω and also provide specific signatures for conducting electron scattering off impurities. It can be calculated from

10 $$\sigma ({\rm{\Omega }})=\frac{-1}{{\rm{\Omega }}}{\rm{Im}}\,[{{\rm{\Pi }}}_{xx}(i{{\rm{\Omega }}}_{n}\to {\rm{\Omega }}+i\delta )].$$

With the Green’s function replaced by its spectrum function, Eq. (10) reduces to be^41, 46^

11 $${\rm{\sigma }}({\rm{\Omega }})=\frac{{(\hslash {v}_{F}e)}^{2}\pi }{{\rm{\Omega }}}{\int }_{-\infty }^{\infty }d\omega [f(\omega )-f(\omega +{\rm{\Omega }})]\sum _{{\bf{k}}}Tr[A({\bf{k}},\omega ){\sigma }_{y}A({\bf{k}},\omega +{\rm{\Omega }}){{\rm{\sigma }}}_{y}\mathrm{].}$$

**Figure 6:**
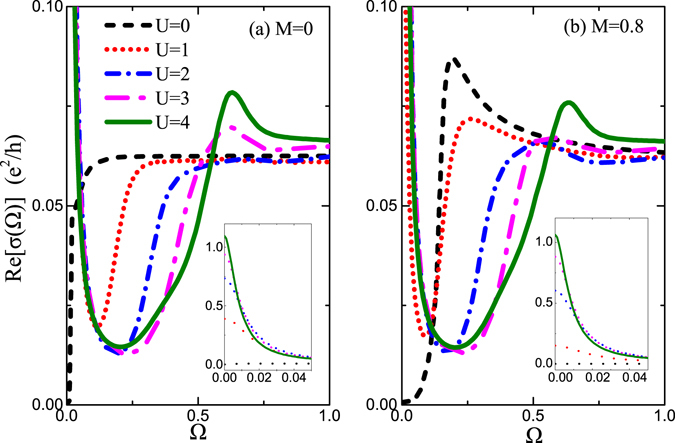
The optical conductivity σ(Ω) as a function of photon frequency Ω for gapless case *M* = 0 (**a**) and gapped case *M* = 0.8 (**b**). Insets are the blowup in the vicinity of Ω = 0 for corresponding main frame. The other parameters are the same as in Fig. 1.

In Fig. 6(a), we show the optical conductivity σ(Ω) for gapless TI surface as a function of positive frequency Ω. The system is set at μ = 0 at the Dirac point and low temperature *T* = 0.01. For undoped case *U* = 0 (black dashed line), the Drude conductivity is zero due to vanished DOS at the Dirac point, but it quickly reaches a steady value as long as Ω slightly deviates away from Ω = 0. This is characteristic of a metallic response when the Fermi energy crosses a band. With the increase of *U*, there develops a Drude conductivity peak, which increases quickly starting from zero, as shown in the inset of Fig. 6(a). Meanwhile, the absorption jump is pushed towards the high frequency. One recalls that the onset of the absorption band comes from the interband transitions which starts at *ω* = 2*μ*. But here, though *μ* = 0 is set, there still exists an effective chemical potential *μ* contributed by impurity-induced self-energy Re[∑_0_(*ω*)]. Thus, the position of jump is located at ${\rm{\Omega }}=2\tilde{\mu }$ which becomes large with increase of *U*. Between the Drude peak and the jump, there appears the Pauli blocked region of the optical response where the optical conductivity is heavily suppressed due to less electron-hole pair excitations. Besides the movement of absorption edge, another profound feature is that just above the jump frequency, there emerges a new conductivity peak. This interband conductivity peak becomes prominent with the increase of potential *U* and can characterize the formation of impurity resonant state. The underlying physics is that the resonant state enhances the absorption of light through the creation of an electron–hole pair. In high frequency limit Ω → ∞ all curves tend to a saturated constant, regardless of the potential strength. This is a typical characteristic of 2D Dirac materials^47^, different from 3D Dirac or Weyl system^41, 46^ where *σ*(Ω) exhibits a linear increase with Ω after the jump.

The dependence of the optical conductivity on photon frequency for gapped case is illustrated in Fig. 6(b), where *M* = 0.8 and the other parameters are the same as in panel (a). Even in absence of *U*, there also appears a jump located at Ω = 2Δ, characterizing the magnetic impurity induced bang gap. Below the jump frequency, the optical conductivity vanishes, corresponding to the gapped regime, but above the jump the optical conductivity exhibits a large peak. With the increase of *U*, the self-energy Re[∑_0_(*ω*)] play a role. In comparison with gapless case, the absorption edge for gapped case moves more slowly for small *U*, e.g., *U* = 1, due to existence of gap. For large *U* = 4, the magnetic gap is almost completely filled and recover the gapless pattern. The corresponding Drude conductivity is shown in the inset in Fig. 6(b), where the value increases slowly first and quickly after gap is filled.

As temperature increases, the slope of absorbtion jump becomes smooth and the low frequency region of the curve is lifted due to the transfer of spectral weight from the interband to the intraband, relieving Pauli blocking in the conduction band and increasing the probability of occupation in the valence band.

## Conclusions

We have presented a comprehensive study of electronic properties on a TI surface in the presence of impurities, with particular attention paid to the role of resonant scattering on impurities. We calculate the dc and ac conductivities, the Seebeck coefficient, and the figure of merit within the full Born approximation in the longitudinal direction. It is found that the dc conductivity at low temperatures is not of linear dependence but shows a zero-bias peak, whose height is sensitively dependent on the scattering potential, different from the constant results in graphene^38, 45^. The underlying physics is that the impurities create the complex energy dependence of self-energy with the formation of resonant state close to the Fermi level. The sharp structure of the impurity resonant state near the Fermi level also modifies significantly the behavior of thermopower, which exhibits an interesting switch from n-type to p-type carriers. In the low energy regime, the resonant state makes the Lorenz number deviate the Wiedemann-Franz ratio, and is manifested by the dip in the figure of merit. The transports through the gapped TI surface are explicitly analyzed, where the filling process of a magnetic gap by the resonant state is featured with remarkable change of thermoelectric signatures. Finally, We further study the impurity effect on the dynamic optical conductivity. It is found that the resonant state also generates a conductivity peak above the absorption edge for the interband transition. The frequency position of the absorption edge can be controlled by the doping as a consequence of impurity-induced breaking of particle-hole symmetry. The thermoelectric transports provide a new approach to probe the impurity effect on TI materials.
